# Unexpected links reflect the noise in networks

**DOI:** 10.1186/s13062-016-0155-0

**Published:** 2016-10-13

**Authors:** Anatoly Yambartsev, Michael A. Perlin, Yevgeniy Kovchegov, Natalia Shulzhenko, Karina L. Mine, Xiaoxi Dong, Andrey Morgun

**Affiliations:** 1 Department of Statistics, Institute of Mathematics and Statistics, University of Sao Paulo, Sao Paulo, SP Brazil; 2 College of Pharmacy, Oregon State University, Corvallis, OR USA; 3 Department of Mathematics, College of Science, Oregon State University, Corvallis, OR USA; 4 College of Veterinary Medicine, Oregon State University, Corvallis, OR USA; 5 Instituto de Imunogenética - Associação Fundo de Incentivo à Pesquisa (IGEN-AFIP), São Paulo, SP Brazil

## Abstract

**Background:**

Gene covariation networks are commonly used to study biological processes. The inference of gene covariation networks from observational data can be challenging, especially considering the large number of players involved and the small number of biological replicates available for analysis.

**Results:**

We propose a new statistical method for estimating the number of erroneous edges in reconstructed networks that strongly enhances commonly used inference approaches. This method is based on a special relationship between sign of correlation (positive/negative) and directionality (up/down) of gene regulation, and allows for the identification and removal of approximately half of all erroneous edges. Using the mathematical model of Bayesian networks and positive correlation inequalities we establish a mathematical foundation for our method. Analyzing existing biological datasets, we find a strong correlation between the results of our method and false discovery rate (FDR). Furthermore, simulation analysis demonstrates that our method provides a more accurate estimate of network error than FDR.

**Conclusions:**

Thus, our study provides a new robust approach for improving reconstruction of covariation networks.

**Reviewers:**

This article was reviewed by Eugene Koonin, Sergei Maslov, Daniel Yasumasa Takahashi.

**Electronic supplementary material:**

The online version of this article (doi:10.1186/s13062-016-0155-0) contains supplementary material, which is available to authorized users.

## Background

It is quite common, especially in biology, that in order to understand how systems transition from one state to another (e.g. from health to disease) scientists compare how parameters such as gene expressions, protein levels, or metabolite abundances differ between these states. One result of such a comparison is a list of parameters up- or down-regulated (due to the increase or decrease of some numerical value attributed to the parameter) from the first state to the second. In case of gene expression, these alterations represent a consequence of the two key factors: first, the original stimulus (e.g. mutation or environmental perturbation) that underlies the transition of a biological system from one state to another; and the second factor, a biological process that drives regulatory relations between individual genes independently on the presence of the stimulus. In other words, regulatory relations in biological systems (as well as many other systems) are not generally functions of the state but are rather pre-determined by biological roles of the components.

Most frequently, the components like genes are not regulated independently from each other; rather, they make up regulatory networks [1–5]. A common approach and the first step to the reconstruction of regulatory network structure is the inference of a correlation network built from parameters differentially abundant between two states. In particular, correlation (or, for the purposes of this paper, co-variation) networks are widely used in gene expression analysis.

Indeed, gene expression networks have been widely used to advance global understanding of principles that govern regulatory processes in biology [6, 7], to disclose molecular mechanisms of diseases [8], and even helping with finding better drugs [9]. Indeed, medical fields such as cardiology [8], endocrinology [10, 11], immunology [4, 12, 13], host-microbiome interactions [10, 12] and others have benefited from gene network analysis. Cancer is a very good example of applications of gene expression networks because insights from network analyses were essential for identification of key drivers of carcinogenesis for brain [14, 15], breast [15], skin [9] and cervical [16] tumors.

Co-variation network analysis works under the assumption that any edge (link) in a network, corresponding to a correlation between two parameters/nodes, is the empirical result of either direct or indirect (i.e. confounding) causal relationships, unless the edge is erroneously drawn (i.e. the observed correlation is an artifact of statistical error) [17–19]. Thus, we hypothesized that in a co-expression network there may be a relationship between the sign of correlation (i.e. positive or negative) of two regulated genes and the direction of their change between the two states (i.e. up or down-regulation). In this paper, we demonstrated the presence of this inter-dependence in different types of data, found that a departure from this relation reflects a proportion of erroneous edges in the regulatory networks, and developed a mathematical theory of this phenomenon.

## Results

### The concept of unexpected correlations

**Fig. 1:**
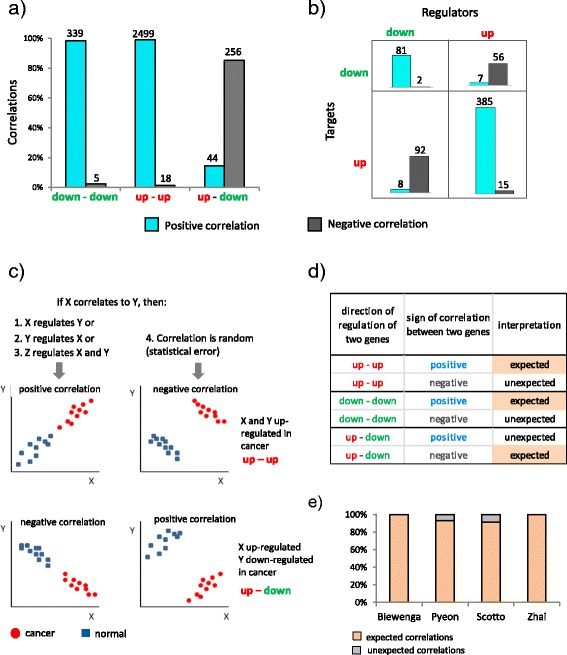
Sign of correlations corresponds to the direction of change in regulatory networks. **a** Percentage of positive and negative correlations for pairs of up-regulated (*up*) and down-regulated (*down*) genes observed in the network from Mine et al., 2013; **b** number of positive and negative correlations between pairs of target and regulator genes in relation to their up- or down- regulation in cervical cancer data; **c** examples of regulatory (*left panels*) and erroneous (*right panels*) connections between genes X and Y; **d** possible combinations of gene regulations and correlations with the interpretation of connection; **e** percentage of expected and unexpected connections between LAMP3 and other differential expressed genes in cervical cancer corresponding to genes regulated after knockdown of LAMP3 in four datasets: Beiwenga (GSE7410), Pyeon (GSE6791), Zhai (GSE7803), Scotto (GDS3233)

In order to verify whether there is a relationship between the direction of gene regulation and the sign of correlation we used a gene co-expression network from our recently published paper on network analysis in cervical cancer [16]. We felt that this network should provide excellent real data for this analysis, as it was constructed from a robust meta-analysis of five cancer gene expression datasets (GSE26342, GSE7410, GSE9750, GSE6791, GSE7803) and thus validated by large, independent sources. This network contained 738 nodes with 490 up and 248 down-regulated between cancer and normal tissues. These nodes were connected by 3161 edges with 2882 representing positive and 279 negative correlations. Relating these two types of information, we observed a strong association between the direction of gene expression change and the sign of correlation (Fig. 1a). Positively correlated genes in ~98 % cases had concordant increases or decreases in gene expression (up-up or down-down), and negatively correlated ones in ~92 % of cases were regulated in opposite directions (up-down). At first glance we found surprising such a strong association and sought to further evaluate this phenomenon. Thus we focused on a part of this big network, which is a bi-partite network consisting of 626 correlations between gene-regulators and gene-targets [16]. In this smaller network, in which correlation links could more obviously correspond to causal links (because gene-regulators have changed their expression as a result of chromosomal aberrations (Additional file 1: Figure S1)), we found similar association between direction of correlation and gene regulation (Fig. 1b).

We wondered whether such association can be generalized to other gene regulatory systems with two states (e.g. health and disease) and two types of regulation (stimulation and inhibition). In order to further investigate this, we propose a scheme in which we associate the sign of correlation (+/−) of each network edge with the direction (up/down) of gene regulation between system states. Sign association follows a simple set of rules:

- If there is a correlation between two “up” or “down” regulated genes (as in the top left panel in Fig. 1c), the sign associated with the link is positive.
- If there is a link between an “up” regulated gene and a “down” regulated gene (as in the bottom left panel in Fig. 1c), the sign associated with the link is negative.

The full set of possible combinations of gene regulations and correlations are given in (Fig. 1d). We hypothesize that correlations whose sign disagrees with the corresponding association are *erroneous*, i.e. they are the result of statistical error rather than causal relationships; or, they can be the results of an external/indirect influence, which is irrelevant for transitions between the biological system states. We will hereafter call such correlations *unexpected* (Fig. 1d), and their proportion among all correlations in a network is abbreviated as PUC (Proportion of Unexpected Correlations).

Since the original observation (Fig. 1) was made in complex system we also wanted to test the association between the sign of correlation and the direction of change in gene expression in the system where cause of gene regulation can be unambiguously defined. For this, we employed a basic principle claiming that a result of experimental perturbation represents a *bona fide* causality relationship. In the same cervical cancer work, we had performed siRNA perturbation of gene LAMP3 (GSE29009), which was one of the key gene-drivers of the antiviral subnetwork. Our theoretical prediction would be that genes whose expression is affected by perturbation of the gene-driver (i.e. LAMP3) *in vitro* and correlated to the expression of the gene-driver in the original cancer data should present correlations of the expected sign. For example, if a gene was down-regulated by LAMP3 siRNA, it is expected to be positively correlating to LAMP3 in the cancer gene expression data and vice versa (i.e. if gene is up after siRNA treatment correlation should be negative). Thus we analyzed if the direction of regulation of genes affected by LAMP3 siRNA in the cell line was corresponding to the sign of correlation between each gene and LAMP3 in four cervical cancer datasets (GSE7410, GSE9750, GSE6791, GSE7803). In these datasets, we observed that almost all correlations between LAMP3 and genes whose expression was affected by LAMP3 siRNA had correlation signs concordant to the directions of gene regulations due to siRNA treatment (Fig. 1e). Thus, this data provides the additional experimental support for our hypothesis about non-random interdependence between sign of correlation and direction of gene regulation.

### Mathematical formalism

Encouraged by these results, to better understand the properties of this new metric (PUC) we went further to establish a rigorous mathematical framework.

Our hypothesis that unexpected correlations are erroneous can be rigorously proven for systems that transition between two stable states with two types of relationship between parameters: stimulation and inhibition. Herein, as an example, we provide a proof of our hypothesis using a simple Bayesian network^9^ with two equilibrium states and linear dependences between nodes. The general case is considered in Section II.2 of the Additional file 1.

In order to formulate our results, we begin by stating the following mathematical notions and definitions. A regulatory network is represented as a directed acyclic graph (DAG) *G* = (*V*, *E*). Any edge *e* ∈ *E* is an ordered pair of vertices (nodes) *e* = (*v*, *w*) ∈ *V* ^2^. The order of vertices in an edge represents the direction of causality in a regulatory network (that is, in the edge (*v*, *w*), *v* regulates *w*). For any node *v* we associate the set of its parents as *pa*(*v*) := {*u* ∈ *V* : (*u*, *v*) ∈ *E*}. We define the set of root-nodes *gf*(*G*) for the graph *G* as the set of all nodes without parents: *gf*(*G*) := {*v* ∈ *V* : *pa*(*v*) = ∅}. For simplicity, we consider a regulatory network with only one root-node, |*gf*(*G*)| = 1, denoted by the vertex *o*. The case with more than one root-node is covered by the general model considered in Additional file 1, see SectionI.et graph *G* be weighted, meaning that every edge *e* = (*v*, *w*) ∈ *E* has an associated label (weight) *c*_*vw*_ ∈ ℝ. With every node *v* ∈ *V* we associate a random variable *M*_*v*_. Variables *M*_*v*_, *v* ∈ *V*, are connected by the following structural linear equations:

1 $$ {M}_v={\displaystyle \sum_{w\in pa(v)}}{c}_{w v}{M}_w+{\varepsilon}_v. $$

Here, the random variables *ε*_*v*_, *v* ∈ *V*, representing the noise in the system, are mutually independent and identically distributed with mean 0 and variance *σ*_*v*_^2^. We suppose heteroscedasticity with uniformly bounded variances: there exists *σ* ^2^ such that *σ*_*v*_^2^ ≤ *σ* ^2^ for all *v* ∈ *V*. By defining the distribution of the root-node variable *M*_*o*_, we obtain a unique joint distribution of random variables *M*_*v*_, *v* ∈ *V*. This joint distribution will be referred to as *equilibrium state*.

In the previously discussed biological framework, a graph *G* represents the entire gene expression network. A node *v* represents a gene with the corresponding expression level *M*_*v*_. . An edge *e* = (*v*, *w*) represents a causal link between two genes *v* and *w* in which the expression of *w* is regulated by *v*, and *c*_*vw*_ is the interaction weight. The sign of *c*_*vw*_ reflects the direction of regulation: a negative (positive) sign corresponds to inhibition (stimulation). The parents of *v* are simply all genes which regulate *v* and the root-node of *G* is the primary regulator of the entire network.

In order to define two distinct equilibrium states, say *P* and *Q* (e.g. case and control, disease and health, etc.) for a system defined by causal graph *G* and structural equations (1), we need only to define two independent root-node variables, *M*_*o*_^(*P*)^ and *M*_*o*_^(*Q*)^, together with mutually indepennt noise variables *ε*_*v*_^(*P*)^, *ε*_*v*_^(*Q*)^, *v* ∈ *V*. Let *M*_*v*_^(*P*)^ and *M*_*v*_^(*Q*)^ denote the expressions of the gene at node *v* in two distinct equilibrium states *P* and *Q*. For any *v* we denote the changes in expression between states as ${\varDelta}_v=\mathbb{E}\left({M}_v^{(P)}\right)-\mathbb{E}\left({M}_v^{(Q)}\right)$, where $\mathbb{E}$ denotes the expectation value (mean) of corresponding variable.

The mathematical definition of expected and unexpected links, as introduced informally in the introduction, is now formally expressed in the following definition.

**Definition** *. An edge e* ∈ *E is called an expected link between nodes v*, *w* ∈ *V if and only if Δ*_*v*_ *Δ*_*w*_ *cov*(*M*_*v*_^(*P*)^, *M*_*w*_^(*P*)^) > 0 *and Δ*_*v*_ *Δ*_*w*_ *cov*(*M*_*v*_^(*Q*)^, *M*_*w*_^(*Q*)^) > 0*. An edge which is not an expected link is said to be an unexpected link.*

This definition effectively states that the directions of regulation of two genes between two states should agree with the sign of the correlation between them within each state.

Note that the covariances in the definition can be substituted by the coefficient of correlation (Pearson correlation).

In the main lemma stated below, we show that here, all unexpected links are produced by the noise in the system: i.e. if *σ* ^2^ is small enough, then the system will have no unexpected links.

*Lemma 1. For any finite DAG with linear structural equations (1) and two equilibrium states there exists some σ*_0_ *such that if σ*_*v*_^2^ < *σ*_0_^2^ *for all v* ∈ *V, then there are no unexpected links in the system.*

The proof is given in Section II.1 of the Additional file 1. Another very important property of the concept of unexpected links is that PUC represents and identifies approximately half of all erroneous correlations:

$$ 2\mathbb{E}(PUC)\approx \mathbb{E}\left(\mathrm{total}\ \mathrm{proportion}\ \mathrm{of}\ \mathrm{false}\ \mathrm{positive}\ \mathrm{links}\right)\ (2). $$

A formal proof of this statement (under certain conditions) is given in Section III.3 of the Additional file 1, as well as an explanation for why this makes intuitive sense. The basic idea is that false edges are, in principle, equally likely to have expected correlations as they are to have unexpected correlations.

### Unexpected correlations reflect the noise in real and simulated networks

Lemma 1 shows that in regulatory networks unexpected correlations must have appeared as a result of noise within the network and that the proportion of unexpected correlation thus reflects the noise level in a network.

**Fig. 2:**
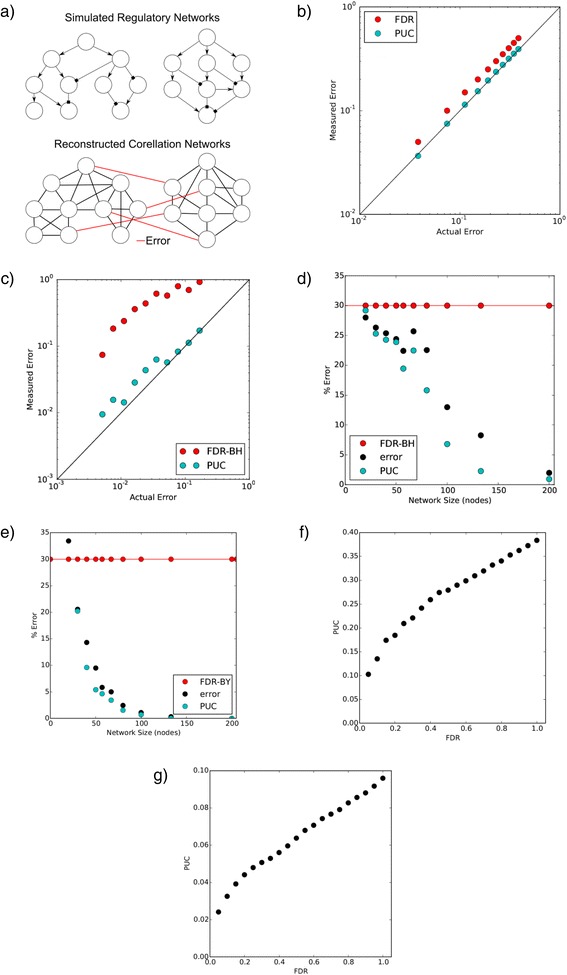
Comparison of PUC and FDR. **a** Two regulatory networks are simulated independently, then both networks’ node expression levels combined into one data set. In a correlation network constructed from the simulated data, any correlations (links) between nodes from independent networks are known to be erroneous; Bayesian simulations (**b**), as well as gene regulatory simulations performed GeneNetWeaver (**c**) suggest that PUC more accurately reflects network error than FDR (Benjamini-Hochberg, FDR-BH); as network size grows, PUC more accurately reflects network error than FDR-BH (**d**) or its variation with multiple hypothesis under dependence called FDR Benjamini-Yekutieli (FDR-BY) (**e**); PUC correlates with FDR in both gene expression (**f**) and macroeconomic (**g**) data

Mathematical models are restricted by the domain of their assumptions, which limits their applicability. Thus, although we have empirically observed a small PUC in a high confidence cervical cancer network (Fig. 1a-b), we wanted to verify whether this correspondence would still hold in different settings. We therefore analyzed 24 additional data sets retrieved from the BRB Array Tools Archive (see Additional file 1) providing gene expression network transitions in different types of cancer, and found that PUC strongly correlated with FDR (correlation coefficient of 0.87 CI95% 0.8117–0.9416). Turning our attention to phenomena other than cancer, we also analyzed the gene expression network perturbed as a result of colonization of intestinal tissue with normal microbiota (i.e. the mix of microorganisms that live in the gut). In these data (GSE60568) [13] and again found that PUC is highly correlated with FDR (Fig. 2f).

Thus, the observation of strong correlation between FDR and PUC in multiple datasets from diverse biological settings in two different species (*Homo sapiens* and *Mus musculus*) provides additional support for our prediction that PUC, similarly to FDR, quantitatively reflects network error.

An important question, however, is whether PUC brings any advantage over the standard approach to measuring the proportion of erroneous edges in a reconstructed regulation network (i.e. FDR). Real data makes such a comparison difficult, because though both methods of analysis will return values for network error, there is not necessarily any obvious way to determine which is more accurate; i.e. in real data, the actual regulatory network is not known.

To investigate the behavior of PUC in a “controlled environment” we simulated networks using two approaches. We have used simple Bayesian networks [20] with structural equations (1) and the software GeneNetWeaver [21] which uses ordinary and stochastic differential equations as models for gene regulation. To compare the effectiveness of PUC and FDR, we construct a regulatory network as two distinct disjoint regulatory sub-networks, and gene expressions are simulated independently according two equilibrium states. In an empirical correlation network constructed from the simulated data, any correlations (link) between nodes from distinct sub-networks are known to be erroneous (Fig. 2a). This design allows for a true measure of network error against which to compare PUC and FDR analysis results.

In order to determine which method (FDR or PUC) better quantifies error, we look at all three measures of error (FDR, PUC, and the true error) and compare the accuracies of FDR and PUC. The results of both types of simulations suggest that PUC is more accurate than FDR in estimating true error, although there is a strong correlation between the two metrics (Fig. 2b,c).

The FDR family of methods is the most popular procedure for large-scale p-value correction for multiple hypotheses [22–26]. All these FDR methods, however, ignore the dependence structure between hypotheses, which leads to the fact that FDR is an overly conservative approach (i.e. it overestimates the number of false positives).

In the case of regulatory networks, each edge constitutes a hypothesis; interdependency of regulatory network hypotheses manifests in indirect regulation between genes. Indeed, this is exactly the case with co-variation networks, in which it is possible to find numerous indirect pathways with only a few direct links.

Using PUC as a measure of error, however, does not require any assumption about independence of hypotheses. PUC may thus be more accurate than FDR for error estimation in co-variation networks with a large number of interconnected nodes. The “degree of dependency” between hypotheses also depends on the size and number of sub-networks that compose a network. A network made up of twenty sub-networks consisting of twenty nodes each should have a lower degree of hypothesis interdependency than a single network consisting of four hundred nodes lacking any well-defined sub-networks.

In order to pinpoint this effect we simulated various networks up to 400 nodes in disjoint sub-networks, each with an equal number of nodes (for example, 20 disjoint sub-networks with 20 nodes each). While both types of simulations (Bayesian and GeneNetWeaver networks) showed overall more accurate results for PUC, in Bayesian networks we also observed lower efficiency of FDR for large networks (Fig. 2d). This effect, however, was less pronounced in GeneNetWeaver- simulated networks (Additional file 1: Figure S4). Furthermore, we obtained similar results (Fig. 2e) by using another version of FDR which was designed to correct for hypothesis interdependency – Benjamini-Yekutieli, FDR-BY [27].

### PUC in a non-biological system

The fact that we could mathematically prove the relationship between unexpected correlations and network error suggests that this principle could be widespread beyond gene interactions in biological systems. As a proof-of-concept of PUC’s generality, we turned our attention to economics. The justification for this choice of subject relates to the presumption that economic systems, similarly to biological systems, are governed by cause-effect relationships and can, by extension, be described by regulatory networks. We analyzed 1503 parameters retrieved from World Bank economic databases for the year 2008 in 193 countries in such areas as business, education, health, etc. (details provided in the Additional file 1). Parameters with bimodal distributions defined distinct states of economic networks for any given country. Figure 2f shows PUC for parameter correlation networks with different FDR thresholds in which a particular parameter (expenditure per student on primary education as a percent of GDP per capita) defined distinct network states; Figure S2 (in the Additional file 1) shows similar graphs for various other parameters. As expected, these networks demonstrated a high concordance between the network errors given by PUC and FDR. This result supports the idea that the concept of unexpected correlations can be extended to a large variety of causal networks and that measurement of the proportion of unexpected correlations (PUC) can improve network analysis in a variety of scientific disciplines.

### Estimating error using PUC

The entire procedure of PUC for calculating network error is as such: first, all correlations in a differential expression list are ranked by p-value. A network is constructed with edges consisting of correlations within an arbitrary p-value threshold (e.g. 0.01). Unexpected links are identified, counted, and removed from the network. The final measure of error in the remaining network is given by, where and are respectively the numbers of total and unexpected links in the network prior to removal of unexpected links. The reason for this formula is explained in the last paragraph of mathematical formalism section, and has to do with the fact that the number of unexpected links in a network is approximately equal to half of the total number of false links.

## Discussion

The growth of molecular biology has advanced such that we can measure the expression of thousands of genes simultaneously. Simply measuring the expression of multiple individual genes, however, is insufficient to describe a systems issue such as complex diseases. To relate gene expression to physiological states (e.g. disease) and other variables in an organism’s environment we utilize gene expression networks. These networks enable more intelligent identification of molecular subtypes of diseases and molecular targets for treatment. The reconstruction of gene expression networks, however, is not easily accomplished. Constructing reliable gene expression networks with current methods requires obtaining large data sets because a large number of hypotheses are required to be tested for network inference.

Although the False Discovery Rate (FDR - Benjamini-Hochberg) is the most popular multiple hypothesis correction method, its application for network inference is a conservative procedure and makes the often unfitting assumption of the independence between correlations in gene networks. There are less popular versions of FDR, for example Benjamini-Yekutieli [27], which take into account various dependence structures between the hypotheses under consideration, but the usage of this did not demonstrate any significant advantage over PUC (see Fig. 2e). Consequently, these corrections tend to have a high rate of false negative discovery (i.e. low power) and require vast sample sizes in order attain desirable degrees of certainty about reconstructed networks. There is thus a critical need for more powerful methods of estimation of false positive connections between genes in co-expression networks.

In this study we have revealed and mathematically proved a new feature of co-expression networks. This feature is based on the natural notion that any correlation has direct or indirect causal components and noise components. In the case when causal components prevail over noise, the sign of a correlation between two genes should be related to their up- or down- regulation of the genes between two states (Fig. 1). We first observed this relation empirically in gene expression datasets [16, 28], and subsequently in macroeconomic data (see Fig. 2f and Additional file 1: Figure S2). The observation of this network feature (relation between sign of correlation and direction of change) in data of such a different nature (biology and economics) suggests that this relation is a universal property of covariation networks.

We proposed using this relation for identifying false connections in co-variation networks increases network accuracy and estimates network error. This approach demonstrates clear advantage over the classic method (FDR) not only by providing better estimates of error in large co-variation networks, but also by allowing the removal of approximately half of all erroneous edges.

The identification of unexpected correlations has two primary impacts. Firstly, it provides a new method to estimate the proportion of erroneous links in a network. Secondly, it allows for the *removal* of approximately half of the erroneous edges in the network (namely, those that are unexpected), decreasing their proportion by a factor of two and thereby improving the overall accuracy of the reconstructed network.

The concept of expected and unexpected correlations that we introduced is closely related to the concept of monotone causal effects and the covariance between them [29]. Where the authors proved (see the theorem 4 in the paper [29]) that the covariation between any two positively (negatively) monotonically associated variables is non-negative (non-positive). It corresponds to our definition of expected correlations. Lemma 1 we proved for linear relations should therefore hold for any monotone relationships; this idea is expanded in Section II.2. of the Additional file 1, and the framework of PUC extended to a broader class of networks than those mentioned thus far.

It is also important to further investigate how non-monotonicity affects the notion and application of unexpected correlations. The concept of non-monotonicity can be exemplified for our problem as different types of relationships in two network states, such as a negative correlation between parameters in one biological state and a positive correlation in another. In such cases, despite violation of monotonicity, we expect unexpected correlations to arise primarily due to noise, rather than the change in relationships. Nonetheless, we demonstrated (see Section II.4. of the Additional file 1) that there is no evidence for non-monotonicity to suggest that these exceptionally rare non-erroneous correlations are in fact responsible for the observed changes in gene expression between states of a biological system. Therefore, because the ultimate goal of network inference is actually to model and understand the transition of biological system from one state to another, we can safely remove these unexpected correlations from the reconstructed network for independent reasons (i.e. that they do not have causal contribution to system state transition).

We believe that this work, besides revealing a new feature of co-variation networks, introduces an entirely new way of dealing with error in their reconstruction. Indeed, statistical methods employed for such problems normally estimate an error, but cannot detect erroneous edges. We propose a method that besides (according to simulations, potentially superior) error estimation allows for identification and removal of approximately half of total network error. Thus, the identification and removal of unexpected correlations decreases the proportion of irrelevant and erroneous connections and strongly increases the power of network inferences.

## Conclusion

This study reports a discovery of a new property of interdependence between sign of correlation and direction of gene regulation for covariation networks first observed by us in cervical cancer. It appears to be universal as it has been further found in wide range of phenomena within biology and economics. Furthermore, the newly revealed property provides a basis for developing a method for measuring the proportion of erroneous edges in a network. This method stands out among standard approaches like the false discovery rate (FDR), because besides estimating an error it allows for the elimination of about half of all incorrect links in a network under a given statistical threshold.

## Reviewers’ comments

### Reviewer’s report 1: Eugene Koonin, NIH

Reviewer comments:

In this paper, Yambartsev and colleagues develop a new approach to the analysis of covariation networks that is specifically applied to networks of gene co-expression from cancers and normal tissue controls. They make a straightforward observation that to me is quite intuitive, namely that there is a link between the sign of correlation between between expression profiles of a pair of genes and the directionality of their regulation between the compared states. In other words, if in a pair of genes, both are either down-regulated or up-regulated in cancer compared to normal tissue, they are expected to show a positive correlation. Conversely, if the directions of the regulation in a pair of genes, are different, they will show a negative correlation. The authors demonstrate the validity of this connection on experimentally characterized examples and simulated networks and prove analytically that such a connection should exist. They then employ this link to introduce a very simple but apparently powerful metric for measuring noise in covariation networks, namely PUC (proportion of unexpected connections), i.e. the fraction of edges in a network that violate the above rule. Remarkably, the PUC appears to perform significantly better than FDR. As far as I can see, the approach developed in this work can become important in the analysis of covariation networks, especially in the context of the comparison between different states (disease vs normal, normal vs stressed etc.) which is becoming increasingly important.

*Reviewer question/comment and authors’ response:*

- The two most important ones seem to be the description of the comparison of PUC vs FDR and its statistical significance and the description of the networks and correlations themselves.
  Authors’ response: *We agree that this is a very relevant question that will be answered in our future studies. However, in our simulation results we see so striking differences that statistical significance is obvious (Fig.* 2 *b,c). Furthermore, despite we performed two types simulations (that are considered current standards in the field) it is not clear to what extent these results can be extrapolated to real biological systems. Therefore, we started a new investigation that attempts to disclose which properties of biological system are required for PUC to outperform FDR. As suggested by reviewer, this investigation actually involves statistical comparison between FDR and PUC.*
- Although less critical, I think it is highly desirable to expand the Background section to provide an adequate background on network analysis for cancer and other disease states.
  Authors’ response: *We added the text in the introduction devoted to usage of gene expression networks in biology and biomedical research.*

### Reviewer’s report 2: Sergei Maslov, University of Illinois at Urbana-Champaign

Reviewer comments:

The manuscript describes a statistical method for filtering spurious edges in co-expression networks and thus estimating and improving the overall quality of the network. The main idea of the method is simple and seems to be correct as long as expression samples could be subdivided into two principal subgroups capturing the vast majority of expression variability (e.g. cancerous vs. normal tissues in the example used in the manuscript). In this case one expects the majority of positively co-expressed edges to connect genes that both went up or down in cancer-vs-normal comparison, while negatively co-expressed edges to connect genes that change in the opposite directions. This is true as long as the system has only two main attractor states. What is missing from the manuscript in my opinion is the rigorous discussion of conditions when this two-state model holds and when it does not. How does one separate spurious edges caused by statistical fluctuations from bona fide biologically meaningful gene- gene interactions in attractor states other than the one considered important by the authors of the expression collection (e.g. Comparison of gene expression in cancer vs. normal tissues)? In other words, the Eq. 1 indicates that the method focuses on the eigenvector corresponding to a single (the largest?) eigenvalue of the correlation matrix c_wv, while classifying other potentially biologically meaningful eigenvalues and eigenvectors as noise. I think that authors should spend more time discussing this major limitation of their approach.

*Reviewer question/comment and authors’ response:*

- What is missing from the manuscript in my opinion is the rigorous discussion of conditions when this two-state model holds and when it does not.
  Authors’ response: *Thanks for the question. We made modifications to the text to clarify the notion of equilibrium states in our paper.*
*In the paper we model a system as a Bayesian probabilistic network on directed acyclic graph (which represents causal relation between parameters) and with fixed structural equations. The joint distribution of parameters (i.e. equilibrium state) is defined by the distribution of noise variables (it is a product of distributions with bounded variances) and the distribution corresponding to causal root-nodes. Thus, having two distinct distributions on root-node, we will have two equilibrium states of a system.*
*In the section “Mathematical formalism” we introduced the notion of equilibrium state more explicitly. We are sure that it is possible to generalize the notion of expected and unexpected links for the case of multi-state systems, but our scope here is only two-state systems. The two-states systems are very popular and commonly accepted in biology for example case–control studies.*
*The notion of “equilibrium state” can refer also (as the notion of attractor) to some (stochastic) process in time. But it is possible to interpret an equilibrium state as an invariant distribution of this stochastic process. Changing some parameters of process we can change an invariant distribution, obtaining two or more equilibrium states. Moreover, we can deal with invariant distribution (equilibrium state) without considering directly an underlying process.*
- How does one separate spurious edges caused by statistical fluctuations from bona fide biologically meaningful gene- gene interactions in attractor states other than the one considered important by the authors of the expression collection (e.g. Comparison of gene expression in cancer vs. normal tissues)?
  Authors’ response: *Lemma 1 showed that in simple mathematical models (Bayesian networks defined on DAG with one root-node with monotone structural relations) all unexpected links are generated by a noise. The same is true for more general mathematical models, when the distribution of parameters satisfy some monotone relations. In practice, in rare occasions the unexpected correlations may appear as a result of true biological gene-gene interactions. However, according to Lemma 4 (see Section II.4 in Additional file* 1 *) those rare interactions would not contribute to changes in gene expression observed between two states.*
- In other words, the Eq. 1 indicates that the method focuses on the eigenvector corresponding to a single (the largest?) eigenvalue of the correlation matrix c_wv, while classifying other potentially biologically meaningful eigenvalues and eigenvectors as noise. I think that authors should spend more time discussing this major limitation of their approach.
  Authors’ response: *We would like to study possible extensions of our work, and really we want to discuss more about limitations of the approach. We plan to study how the structural equation can contribute in the existence of unexpected links for example through eigenvector of corresponding matrix of structural equations. But we wanted to fix our first step with the simplest cases considered in the paper.*
- A minor comment: on line 46 of the same page as Eq. 1 authors use EM as opposed to a more traditional notation E(M) to denote the expectation value of M. Both notations are potentially confusing as just a few lines below E denotes the set of edges in the network. I recommend switching to or M_bar to denote the mean value.
  Authors’ response: *We thank the reviewer and we fixed the notations of expectation using suggested traditional ones.*

### Reviewer’s report 3: Daniel Yasumasa Takahashi, Princeton University

Reviewer comments:

This is a well written and very creative work that I enjoyed reading. The authors introduce a new concept called expected/unexpected link to infer which links on a network can be the result of noise (i.e., independent of the phenomena of interest). The idea is quite simple and elegant: If the relationship between the nodes is monotonic, the sign of the changes of the values associated to pairs of nodes in two different conditions should be the same. The assumption of monotonicity is quite general, at least comparing to most of the assumption in any network analysis of biological data, e.g., linear relationship. Therefore, the proposed method is expected to be quite useful and general. The application on cervical cancer network that motivates the article is very convincing. The idea of using siRNA experiment to validate the findings is excellent. The mathematical model and the proof of the claims seem to be correct to best of my understanding. The comparison with FDR is quite striking and this section alone should be enough to motivate people to look into the proposed method (myself included). The proposed method has the potential to become a landmark tool in network analysis.

*Reviewer question/comment and authors’ response:*

I have only minor comments:

1. On Fig. 1e, it would be insightful to also show the proportion of expected/unexpected correlations between LAMP3 and the genes whose expression was NOT affected by LAMP3 siRNA. Given that the article is about false positive control and not so much about false negative control, the figure I am proposing could be in the Additional file 1.
  Authors’ response: *We understand the interest reviewer to evaluate what happens with genes not affected by siRNA treatment. The problem with the request to “*show the proportion of expected/unexpected correlations between LAMP3 and the genes whose expression was NOT affected by LAMP3 siRNA*” is that in order to define correlations as expected or unexpected they have to be regulated. Therefore, if there is no regulation of given gene (by siRNA) we cannot classify correlation between this gene and LAMP3 in any category.*
2. The title for the subsection “Mathematical formalism relating causation and the sign of correlation” is misleading as most people would associate causation to direct links in a network. Probably something like “Mathematical formalism relating causal propagation and the sign of correlation” should be more adequate, as the authors use the word “causal propagation” later in the proofs.
  Authors’ response: *We agree about misleading and we decided to change the title for simplest “Mathematical formalism”.*
3. In the section “Mathematical formalism relating causation and the sign of correlation”, I recommend the authors to try to give concrete and biologically motivated examples for the concepts that they introduce. That will make this section more readable and interesting. For example, what is the meaning of a weight Cvw? What is the meaning of two equilibrium states? The authors can say explicitly that in their cervical cancer example, states P and Q represent normal and cancer, for example. The authors also can say that the assumption of equilibrium states implies that the effect of perturbation in some subnetworks had time to propagate to the entire network. In eq (1) what does mean that the variance is small?
  Authors’ response: *We revised this section adding interpretation of the weights as force of interactions between genes and adding the definition of the notion of equilibriums state as a joint distribution of parameters (gene expressions). We hope that in revised version it is more clear that the perturbation variables in structural equations (1) correspond to the internal noise in the system. The lemma says that if the internal noise is small enough (i.e. the variance of the perturbed terms is small) then there are no unexpected links in the system, it means, in our interpretation, that the unexpected links appear in these systems as a result of a noise.*
4. The authors should discuss in more detail the work by VandeWeele and Robins (2010) in the Discussion of the main text. Currently the reference only appears as a note in the Additional file 1.
  Authors’ response: *The work by VandeWeele and Robins (2010) has the reference number 20 in the main text. We added a comment about this work in discussion. They proved that if the structural equations satisfy strong monotone conditions then it is possible to give a sign for a link: positively monotonically associated variables have positive correlation and negatively – negative correlation. Essentially the authors deal with expected correlations. They showed also that if the strong monotone condition will be substituted by weaker monotone condition then the rule of signs does not hold in general. In our case the monotone conditions appears from the comparisons of mean values, and there are the possibility to have unexpected links. Our aim is, starting from the definition of expected links, to prove the noise source of the appearance of unexpected links. Thus we prefer to mention the work instead of deep discussion of their results.*
5. First paragraph in “Additional file 1, II.3. PUC represents 50 % of erroneous” - I think that the sentence “… are random such that … ” should be changed to “… are mutually independent such that …”.
  Authors’ response: *We decided to maintain the joint distribution of perturbed variables as it is. This noise distribution is a result of the noise propagation in a system, and it cannot be considered as a product distribution, because of nonzero covariances between them. The condition we posed on this distribution is: the half of their correlations of noise variables should be positive asymptotically.*
6. The authors should improve the quality of Additional file 1: Figure S1.
  Authors’ response: *Done*
7. The authors might want to provide a simple computational program to run the proposed method on real or simulated data sets. That might help not only to popularize the method, but also to clarify the idea for some readers.
  Authors’ response: *We add the algorithm in Additional file* 1 *and accompanying text with comments.*

## Additional file

Additional file 1: Supporting results. (DOCX 1036 kb)
