# Supplementary material for: Unexpected links reflect the noise in networks
Source: Biol Direct. 2016 Oct 13;11:52. doi: 10.1186/s13062-016-0155-0 (PMC5480421; doi:10.1186/s13062-016-0155-0)
Supplement: Additional file 1: — Supporting results. (DOCX 1036 kb) [file 13062_2016_155_MOESM1_ESM.docx]

**Additional file 1**

**Unexpected links reflect the noise in networks**

**Authors:** Anatoly Yambartsev^1^, Michael A. Perlin^2^, Yevgeniy Kovchegov^3^, Natalia Shulzhenko^4^, Karina L. Mine^5^, Xiaoxi Dong^2^, Andrey Morgun^2^.

**I. Experimental procedures**

**I.1. Statistically significant correlations between differentially expressed genes (DEGs) and show expected signs**

In our recent study (Nature Commun. 2013;4:1806) we have shown that key drivers of cervical carcinogenesis are located in regions of frequent chromosomal aberrations and that these genes cause most of the alteration in gene expression in cervical cancer. Therefore, in order to evaluate whether statistically significant correlations between DEGs which result from known causal relations follow our prediction we performed the following analysis:

First, we selected two groups of genes from DEGs discovered in our previous study: 1) genes in which it has been determined that chromosomal aberrations are responsible for the change in regulation; and 2) genes located in regions in which aberrations are rare, defined by FqG – FqL between -0.1 and 0.1 (Figure S1). Next, we analyzed gene co-expression in tumors samples in order to find correlations between those two groups of DEGs. We found 626 correlated gene-gene pairs with FDR 5%. We used data from the following datasets for our meta-analysis (performed as described in Nature Commun. 2013;4:1806): GSE26342, GSE7410, GSE9750, GSE6791, GSE7803. In brief, we calculated correlations within the tumor samples of each dataset. If correlations presented the same sign in all datasets, then we calculated a corresponding Fisher meta-analysis p-value. We then computed the FDR for these correlations. The results provided support to our hypothesis that significant correlations should have “expected” signs. Indeed, 95% (594 of 626 total pairs) of significant correlations had expected signs.

**I.2. PUC correlates with FDR in macroeconomic data**

The macroeconomic data we analyzed was a combination of all data for the year 2008 on official UN member states in the following World Bank databases: Doing Business, Education Statistics, Gender Statistics, Health and Nutrition Population Statistics, IDA Results Measurement System; Poverty and Inequality Database, World Development Indicators and Global Development Finance. From this data set, we removed all duplications of macroeconomic parameters, as well as all parameters for which data only existed for $\leq25$ countries. Of the remaining parameters, we used a dip test to determine those which were non-unimodal with a p-value of $<2.2\times{10}^{-16}$. From the resulting set of parameters, we selected several with bimodal distributions, each of which we used to define two distinct states of a macroeconomic parameter network. We then computed PUC for parameter correlation networks at different FDR thresholds using each of these definitions. The results of these calculations are shown in Figure S2.

| (a)  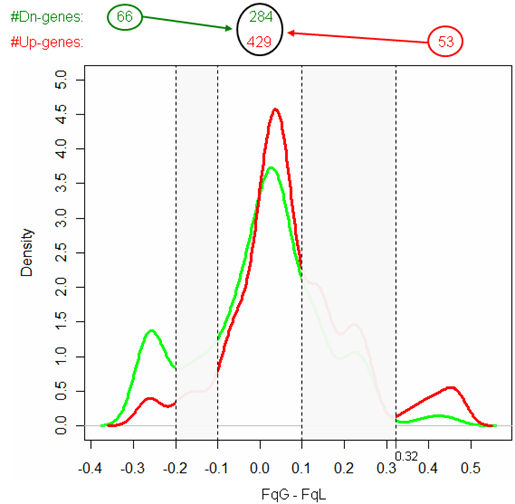 | 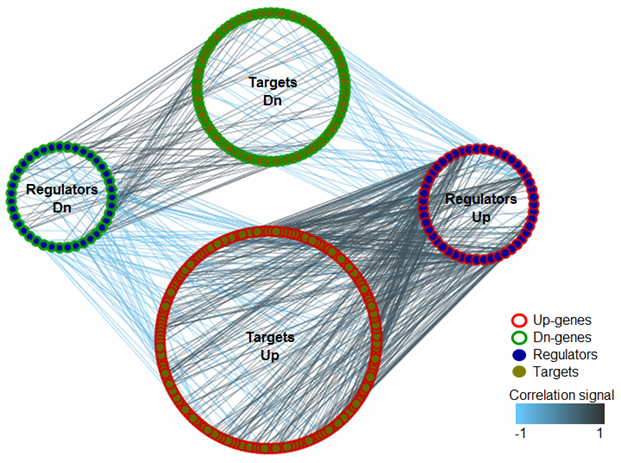(b) |
| --- | --- |
| **Figure S1: Genes directly regulated by chromosomal aberrations can also in turn regulate genes located outside of the aberrations.** (a) Genes regulated by chromosomal aberrations in the expected direction (located in the regions $FqG-FqL< -0.2$ or $FqG-FqL>0.3$) were considered as potential regulators, and genes located within the regions of very rare aberrations ($\left\vert FqG-FqL \right\vert\leq0.1$) were considered to be potential targets. The green (red) line represents up-regulated (down-regulated) genes. (b) The reconstructed regulatory network with correlations in agreement with gene expression. The two green (red/purple) circles are made of up down-regulated (up-regulated) nodes, the middle (side) circles are made up of targets (regulators), and the black (cyan) lines represent positive (negative) correlations.    **Figure S2: PUC and FDR correlate strongly when reconstructing macroeconomic networks using various bimodal parameters to define system states.** Parameters shown: ADA - Duration of compulsory education; AIA - Cause of death, by communicable diseases and maternal, prenatal and nutrition conditions (% of total); AVS - Manufactures exports (% of merchandise exports); BEG - Educational expenditure in pre-primary as % of total educational expenditure; QZ - Private credit bureau coverage (% of adults); RW - Strength of legal rights index; UU Passenger cars (per 1,000 people) | |

**II. Theoretical basis.**

Here we provide some formal definitions of concepts used in the paper and all necessary proofs. This section consists of four parts: 1) we introduce the mathematical machinery for PUC using Bayesian networks; 2) we generalize the previous formalism to handle a broader set of cases; 3) we demonstrate that PUC reflects half of total network error; and 4) we address concerns with network non-monotonicity.

**II.1. PUC on Bayesian networks.**

In order to apply the new concept of noise estimator we use Bayesian Networks as a convenient model for gene expression. Let $G=(V,E)$ be some network, which is directed acyclic graph (DAG). Any edge $e\in E$ is an ordered pair of vertices $e=(v,w)$: and direction of edge is from the first vertex $v$ to the second vertex $w$. We assume that the graph is weighted graph – any edge $e=(v,w)$ has its labels (weight), $c_{vw}$ , which is some real number $c_{vw}\mathbb{\in R}$ . For any node $v$ we associate the set of parents of the node $v$:

$pa\left( v \right):=\left\{ w\in V:(w,v)\in E \right\}$ ( 1 )

We define the set of root-nodes for the graph $G$:

$gf\left( G \right):=\left\{ v\in V:pa\left( v \right)=\emptyset\right\}$ ( 2 )

With any node (gene) $v\in V$ we associate the random variable (gene expression) $M_{v}$. The random variables satisfy the following linear relations (structure equations): for any $v\notin gf(G)$

$M_{v}=\sum_{w\in pa\left( v \right)} c_{wv}M_{w}+\varepsilon_{v},$ ( 3 )

where $\varepsilon_{v}$ are i.i.d. random variable (intrinsic noise) with mean 0 and variance $\sigma^{2}$. Moreover, for simplicity we suppose that there exists only one grandfather $\left| gf(G) \right|=1$ and let us denote it as a vertex $o$.

A path $\pi(v,w)$ of length $n$ from a vertex $v$ to a vertex $w$ is a sequence of edges $e_{i}=\left( v_{i}, v_{i+1} \right), i=1,\ldots,n-1$, with $v_{1}=v$ and $v_{n}=w$. The weight of the path $W(\pi\left( v,w \right))$ is the product of weights of edges from this path:

$W\left( \pi\left( v,w \right) \right):=\prod_{i} c_{v_{i}, v_{i+1}}$ ( 4 )

Let $\Pi(v,w)$ be the set of all paths connecting nodes $v$ and$w$. And let

$W\left( v,w \right):=\sum_{\pi\in\Pi(v,w)} W\left( \pi\left( v,w \right) \right)$ ( 5 )

The graph coupled with expressions we consider as a model of regulatory signaling paths system. The distribution of expressions within the system is determined by the topology of the graph, weights and the distribution of expressions of root-nodes.

For example, let $o$ be the root-node vertex and $M_{o}^{(P)}$ and $M_{o}^{(Q)}$ its expressions in these two different states. Denote by $d^{2}, d$ the variance and standard deviation for root-node expression in two states, and suppose that they do not depend on the state: $d^{2}\mathbb{:=V}\mathrm{ar}\left( M_{o}^{\left( P \right)} \right)\mathbb{=V}\mathrm{ar}\left( M_{o}^{\left( Q \right)} \right)$. Denote the mean changes in expression of root-node´s gene as $\Delta_{o}\mathbb{=E}M_{o}^{(P)}\mathbb{-E}M_{o}^{\left( Q \right)}$. Expression for any non-root-node vertex $v$ can be expressed for any state $S\in\{P,Q\}$ by the formula:

$M_{v}^{(S)}=M_{o}^{(S)}W\left( o,v \right)+\sum_{w\in V\backslash o} \varepsilon_{w}^{(S)}W\left( w,v \right)$ ( 6 )

The mean change in the expression of a gene $v\in V\backslash o$ is given by:

$\Delta_{\boldsymbol{v}}\mathbb{:=E}M_{v}^{\left( P \right)}\mathbb{-E}M_{v}^{\left( Q \right)}=\Delta_{o}W\left( o,v \right).$ ( 7 )

Moreover, for any $S\in\{P,Q\}$:

$cov\left( M_{v}^{(S)},M_{w}^{(S)} \right)=d^{2}W\left( o,v \right)W\left( o,w \right)+\sum_{v^{'}\in V\backslash o} \sigma_{v^{'}}^{2}W\left( v^{'},v \right)W\left( v^{'},w \right)$ ( 8 )

*Definition. We say that a pair of genes* $v,w\in V$ *satisfy* ***expected correlation inequality*** *if and only if*

$\Delta_{v} \Delta_{w} cov\left( M_{v}^{(P)},M_{w}^{(P)} \right)\geq0, \Delta_{v} \Delta_{w} cov\left( M_{v}^{(Q)},M_{w}^{(Q)} \right)\geq0$ ( 9 )

*If (9) holds then we say that the two gene expressions* $M_{v}^{(P)},M_{w}^{(P)}$ *or* $M_{v}^{(Q)},M_{w}^{(Q)}$ *have* ***expected correlations****. If one or both expected correlations inequalities are not satisfied, we say that* $M_{v}^{(P)},M_{w}^{(P)}$ *or* $M_{v}^{(Q)},M_{w}^{(Q)}$ *have* ***unexpected correlations****.*

Note that in the considered model, by (8) the co-variations in (9) do not depend on a state: $cov\left( M_{v}^{(P)},M_{w}^{(P)} \right)= cov\left( M_{v}^{(Q)},M_{w}^{(Q)} \right)$. This independence means that we can use co-variation only in one state in our definition. In this case the following statement takes place.

*Lemma 1. For any finite DAG network with linear relations between variables there exists some* $\sigma_{0}^{2}$ *such that if* $\sigma_{v}^{2}<\sigma_{0}^{2}$ *for any* $v\in V$*, then there are no unexpected correlations into the network.*

*Proof*. Direct from formulas (7), (8). By definition (9) and by representations (7), (8) we have:

$\Delta_{v} \Delta_{w} cov\left( M_{v}^{\left( P \right)},M_{w}^{\left( P \right)} \right){=\Delta}_{o}^{2}W\left( o,v \right)W\left( o,w \right)\left( d^{2}W\left( o,v \right)W\left( o,w \right)+\sum_{v^{'}\neq o} \sigma_{v^{'}}^{2}W\left( v^{'},v \right)W\left( v^{'},w \right) \right)=\Delta_{o}^{2}d^{2}W^{2}\left( o,v \right)W^{2}\left( o,w \right)+\Delta_{o}^{2}W\left( o,v \right)W\left( o,w \right)\sum_{v^{'}\neq o} \sigma_{v^{'}}^{2}W\left( v^{'},v \right)W\left( v^{'},w \right)$ ( 10 )

Here the first term is necessarily positive and the second can be made arbitrarily small by choice of$\sigma_{v^{'}}^{2}$ small enough for all $v^{'}\in V$. Thus $\Delta_{v} \Delta_{w} cov\left( M_{v}^{\left( P \right)},M_{w}^{\left( P \right)} \right)$ can always be made positive (implying that there are no unexpected correlations) by a choice of a sufficiently small variance$\sigma_{o}^{2}$. This statement is precisely Lemma 1.

The formula (8) shows that any link/correlation between two nodes in a network can be represented as a sum of two parts: *causal* *propagation* from causal node and *noise propagation* part:

$cov\left( M_{v}^{(S)},M_{w}^{(S)} \right)=\underset{causal propagation}{\underbrace{d^{2}W\left( o,v \right)W\left( o,w \right)}}+\underset{noise propagation}{\underbrace{\sum_{v^{'}\neq o} \sigma_{v^{'}}^{2}W\left( v^{'},v \right)W\left( v^{'},w \right)}}$ ( 11 )

Here, it is easy to see that if the root-node variance $d^{2}$ increases, then the causal propagation will determine the sign of the covariance after some threshold. It means that it determines a link to be expected or unexpected.

Moreover, Lemma 1 says that if we observe in such regulation networks (DAGs with linear relationships between variables) unexpected correlations, it means that they appeared as a result of noise propagation within the network. Thus, the proportion of unexpected correlation reflects the noise level in a network (to the extent to which this mathematical framework, or that generalized in Section II.2 below, accurately reflects the system being modeled).

*Note 1. The concept of expected correlations was also observed in VanderWeele and Robins, 2010, as a rule governing the relationship between monotonic links and the sign of covariance between variables.*

*Note 2. The linear relations between variables can be generalized: the expression* $X_{v}=f_{v}\left( \left\{ X_{v'} \right\}_{v'\in pa(v)};\varepsilon_{v} \right)$*, where* $f_{v}$ *is a monotone function, and* $\varepsilon_{v}$ *is internal network noise. If structural functions are monotonic function, then the lemma holds also.*

*Estimation of noise.* Error estimation is based on the following: if two genes belong to two independent subnetworks (see Figure 2a), then the correlation between their respective expression levels has to be equal to 0. Observable correlations, however, can be significantly different from 0 due to noise, in which case the observable correlation is positive (or negative) in roughly 50% of the cases (see formula (22)). On average, then, half of all random correlations between any pair of genes from unrelated subnetworks can be classified as unexpected, as in (9). Thus $2\cdot PUC$ can be used as an estimate of total error.

Moreover, it is possible to prove for tree like graphs that within one network the noise propagation (see the formula (12)) has the same property as stated in formula (22). Indeed, the representation (6) means that any variable $M_{v}^{(S)}$ can be decomposed into the causal component $X_{o}^{(S)}W\left( o,v \right)$ and the noise component $\xi_{v}^{(S)}:=\sum_{w\in V\backslash o} \varepsilon_{w}^{(S)}W\left( w,v \right)$. Then the covariance between $\xi_{v}^{\left( S \right)}$ and $\xi_{w}^{\left( S \right)}$ can be calculated exactly (compare with formula (10))

$cov\left( \xi_{v}^{\left( S \right)},\xi_{w}^{\left( S \right)} \right)=\sum_{u\in V} \sigma_{u}^{2}W\left( u,v \right)W(u,w).$ ( 12 )

If $c_{vw}$ are mutually independent, identically distributed, with positive probabilities for being positive or negative, then the covariance (12) for any $S\in\{P,Q\}$ will be negative approximately in half of all cases.

Note that our results are related to mathematical aspect of the phenomenon. In practice we deals with observations, and we should estimate the corresponding variables such as covariances and mean values. Let

$$X^{\left( P \right)}=\left( x_{ij}^{\left( P \right)},i=1,\ldots, p, j=1,\ldots, n^{\left( P \right)} \right)$$

$$X^{\left( Q \right)}=\left( x_{ij'}^{(Q)},i=1,\ldots, p, j'=1,\ldots, n^{(Q)} \right)$$

be observation matrices for two equilibrium states $P$ and $Q$, where $x_{ij}^{\left( P \right)}$ is the observed expression level of $i$-th gene, $i=1,\ldots, p$, in state $P$ in $j$-th sample, $j=1,\ldots, n^{\left( P \right)}$ and $n^{\left( P \right)}$ is the number of samples we have for the state $P$; and, correspondingly, $x_{ij}^{\left( Q \right)}$ is the observed expression level of $i$-th gene, $i=1,\ldots, p$, in state $Q$ in $j'$-th sample, $j'=1,\ldots, n^{\left( Q \right)}$ and $n^{\left( Q \right)}$ is the number of samples we have for the state $Q$. Denote $\bar{x}_{i}^{\left( P \right)}$ and $\bar{x}_{i}^{\left( Q \right)}$ arithmetical mean of $i$-th gene expression if state $P$ and $Q$ correspondingly. Estimation of mean difference of $i$-th gene, $\Delta_{i}$, between states we denote $\hat{\Delta}_{i}:=\bar{x}_{i}^{\left( P \right)}-\bar{x}_{i}^{\left( Q \right)}$. And denote $r_{ii'}^{(P)},r_{ii'}^{(Q)}$ empirical correlation between gene $i$ and $i’$ in state $P$ and $Q$. The statistical analogy of our definition of expected and unexpected links will be the following.

*Definition. An edge* $e\in E$ *is called an* ***expected link*** *between genes* $i,j\in\{1,\ldots,p\}$ *if and only if* $\hat{\Delta}_{i}\hat{\Delta}_{j}r_{ij}^{(P)}>0$ *and* $\hat{\Delta}_{i}\hat{\Delta}_{j}r_{ij}^{(Q)}>0$*. An edge which is not an expected link is said to be an* ***unexpected link****.*

Moreover, in practice we calculate correlation matrix only for one of the state of main interest. For example, in cancer study, first we choose only differentially expressed genes, i.e. the genes which have statistical significant difference $\hat{\Delta}_{i}$, and calculate correlations using only observation sampled from cancer group. Indeed, in practice if for some genes $i$ and $j$ in a state $P$ the condition $\hat{\Delta}_{i}\hat{\Delta}_{j}r_{ij}^{(P)}>0$ holds true, then extremely rarely cases when the condition $\hat{\Delta}_{i}\hat{\Delta}_{j}r_{ij}^{(Q)}>0$ does not hold in state $Q$. Let the state $P$ be the our main interest state, then the PUC calculation will be the following.

***input***: $X^{\left( P \right)},X^{\left( Q \right)}$;

1. choose a set V of differentially expressed genes $i$: $\hat{\Delta}_{i}\neq0$;

2. calculate correlation matrix with elements $r_{ij}^{(P)}$ for $i,j$ from the set V;

3. choose a set of edges E of statistically significant correlations $(i,j)$: $r_{ij}^{(P)}\neq0$;

let $|E|$ be the number of edges into the set of edges E;

4. choose a set U of unexpected links from the set E: $(i,j)\in E$ s.t. $\hat{\Delta}_{i}\hat{\Delta}_{j}r_{ij}^{(P)}<0$;

let $|U|$ be the number of edges into the set of edges U;

***output***: $PUC=\left| U \right|/|E|$; error$=\left| U \right|/(\left| E \right|-\left| U \right|)$.

**II.2. Definitions and generalization.**

Here we study the concept of unexpected links in a more general framework. The positive and negative correlation inequalities are an active research direction in the field of probability and statistical mechanics. We believe these inequalities will allow us to generalize the concept of unexpected correlations in the PUC method. The following framework connects FKG (Fortuin–Kasteleyn–Ginibre) inequality in Statistical Mechanics to the concept of expected and unexpected links.

Let $\Omega$ be the underlying sample space of a biological system. As an example of a biological system we consider a gene regulatory network, where $\Omega$ represents the set of all possible gene expression configurations. We can suppose that the state space $\Omega$ has an ordering (or partial ordering) “≺” assigned to pairs of its elements.

*Definition*. *A random variable* $X=X(\omega)$ *is said to be increasing if* $\omega\prec\omega'$ *implies* $X\left( \omega\right)<X(\omega')$*. Similarly, a random variable is decreasing if* $\omega\prec\omega'$ *implies* $X\left( \omega\right)>X(\omega')$*. Both types of random variables, increasing and decreasing, are said to be monotone random variables.*

In the field of statistical mechanics and probabilistic combinatory, the FKG inequality (Fortuin–Kasteleyn–Ginibre inequality) explains most of the results involving monotone random variables and monotone (increasing or decreasing) events. It states that for two increasing random variables $X$ and $Y$,

$\mathbb{E}\left( XY \right)\mathbb{\geq E}\left( X \right)\mathbb{E}\left( Y \right)$ ( 13 )

In some applications, such as percolation models, partial ordering of $\Omega$ is sufficient for the FKG to hold (Grimmett, 1999). Many important results in applied mathematics and physics, such as the exact value of critical probability in two-dimensional percolation models, would have been impossible without the FKG inequality.

Let $G = (V,E)$ be a graph (network) with vertices (nodes) $V$ and edges $E$. Nodes $v \in V$ represent the genes. Let $X_{v}(\omega)$ be monotone functions (random variables) assigned to each node $v \in V$. Here $X_{v}$ represents the noiseless gene expressions. In this framework it is convenient represent the state system as a probability measure. Consider two probability measures $P$ and $Q$ over $\Omega$ such that for all $\omega\in\Omega$:

$P(\sigma\in\Omega: \sigma\prec\omega)\geq Q(\sigma\in\Omega: \sigma\prec\omega)$ ( 14 )

Here $P$ and $Q$ correspond to the two states of a biological system. Let us denote, as before,$\Delta_{v}:=\mathbb{E}_{P}\left[ X_{v} \right]-\mathbb{E}_{Q}\left[ X_{v} \right]$. We repeat the definition of expected and unexpected links.

*Definition. We say that random variables* $X_{v}$ *and* $X_{u}$ *modeling gene expressions in a pair of genes satisfy* ***expected correlation inequality*** *if and only if*

$\Delta_{v} \Delta_{u} {cov}_{P}\left( X_{v}, X_{u} \right)\geq0, \Delta_{v} \Delta_{u} {cov}_{Q}\left( X_{v}, X_{u} \right)\geq0,$ ( 15 )

*in which case we say that the two gene expressions* $X_{v}$ *and* $X_{u}$ *have* ***expected correlations****. If one or both expected correlations inequalities are not satisfied, we say that* $X_{v}$ *and* $X_{u}$ *have* ***unexpected correlations****.*

*Lemma 2. If* $X_{v}$ *and* $X_{u}$ *are monotone functions, and probability measures*$P$ *and* $Q$ *satisfy the condition (13), then* $X_{v}$ *and* $X_{u}$ *satisfy expected correlation inequality (or* $X_{v}$ *and* $X_{u}$ *have expected correlations).*

*Proof*. Indeed, if $X_{v}$ is an increasing (decreasing) variable, then $\Delta_{v}\leq0$ ($\Delta_{v}\geq0$). Now, if both $X_{u}$ and $X_{v}$ are either increasing or decreasing the FKG inequality (13) implies non-negative correlations, so that for any state$S\in\{P,Q\}$

${cov}_{S}\left( X_{u},X_{v} \right):=\mathbb{E}_{S}\left[ X_{u}X_{v} \right]-\mathbb{E}_{S}\left[ X_{u} \right]\mathbb{E}_{S}\left[ X_{v} \right]\geq0, \forall u,v\in V,$ ( 16 )

which implies expected correlation inequalities (15).

Similarly, if one of the two variables (i.e.$X_{u}$or$X_{v}$) is increasing while the other is decreasing, the FKG inequality (13) implies non-positive correlations, such that for any state $S\in\{P,Q\}$,

${cov}_{S}\left( X_{u},X_{v} \right):=\mathbb{E}_{S}\left[ X_{u}X_{v} \right]-\mathbb{E}_{S}\left[ X_{u} \right]\mathbb{E}_{S}\left[ X_{v} \right]\leq0, \forall u,v\in V$ ( 17 )

implying (15) hold once again. It proves the Lemma 2. ☐

Next, let $\xi_{v}$ denote the errors for each node $v\in V$. We assume that the random variables $\xi_{v}, v\in V$ are functions over a probability space $\Xi$, independent from any probability measure over $\Omega$, such as $P$ and $Q$. Let $\mu$ be the joint distribution of $\xi_{v}, v\in V$ and $\mathbb{E}_{\mu}\left[ \xi_{v} \right]=0$ for any $v\in V$. The measured gene expression we quantify as a random variable

$M_{v}=X_{v}+\xi_{v}, v\in V,$ ( 18 )

over the product space $\Omega\times\Xi$, and the two different states of a biological system correspond to two different probability product measures, $P \times\mu$ and $Q \times\mu$. Note that for any gene $v$:

$\mathbb{E}_{P\times\mu}\left[ M_{v} \right]- \mathbb{E}_{Q\times\mu}\left[ M_{v} \right]= \mathbb{E}_{P}\left[ X_{v} \right]- \mathbb{E}_{Q}\left[ X_{v} \right]=:\Delta_{v}$ ( 19 )

The following Lemma is an analogous of the Lemma 1 for the general framework.

*Lemma 3. If the variances of errors* $\sigma_{v}^{2}= Var(\xi_{v})$ *are small enough for all* $v \in V$*, then the pairs of measured gene expression* $M_{v}$ *will also satisfy the inequalities (15).* *Thus in the noiseless networks we foresee no unexpected correlations.*

*Proof*. The proof is a direct consequence of the covariance calculation.

${cov}_{S\times\mu}\left( M_{u},M_{v} \right)={cov}_{S\times\mu}\left( X_{u}+\xi_{u},X_{v}+\xi_{v} \right)={cov}_{S}\left( X_{u},X_{v} \right)+{cov}_{\mu}\left( \xi_{u},\xi_{v} \right)$ ( 20 )

By Cauchy-Schwarz inequality

$|{cov}_{S}\left( \xi_{u},\xi_{v} \right)|\leq\sigma_{u}\sigma_{v}$ ( 21 )

the second covariance in (19) can be made so small that the sign of ${cov}_{S}\left( M_{u},M_{v} \right)$ and the sign of ${cov}_{S}\left( X_{u},X_{v} \right)$ will coincide. This proves Lemma. ☐

However in the noisy networks, the expected correlations rule (14) can be violated. Here the fraction of edges $(u,v)$ violating (15) that we call the Proportion of the Unexpected Correlations (PUC) becomes an estimator of the frequency of false edges.

**II.3. PUC represents 50% of erroneous**.

For any$u,v\in V$;$S\in\{P,Q\};$ and$\mu\in\Xi,$ let us assume that the error random variables $\xi_{v}, v\in V$ have the following asymptotic property, ${cov}_{\mu}(\xi_{u},\xi_{v})$ is positive for half of the $\left( \begin{matrix} |V| \\ 2 \end{matrix} \right)$edges$(u, v)$, and negative for the rest of the pairs:

$\lim_{|V|\to\infty} \frac{\#\left\{ \left( u,v \right): {cov}_{\mu}\left( \xi_{u},\xi_{v} \right)>0 \right\}}{\left( \begin{matrix} |V| \\ 2 \end{matrix} \right)}=\lim_{|V|\to\infty} \frac{\#\left\{ \left( u,v \right): {cov}_{\mu}\left( \xi_{u},\xi_{v} \right)<0 \right\}}{\left( \begin{matrix} |V| \\ 2 \end{matrix} \right)}=\frac{1}{2}$ . ( 22 )

If the covariance ${cov}_{S\times\mu}\left( M_{u},M_{v} \right)$ is of a different sign than${cov}_{S}\left( X_{u},X_{v} \right)$ (i.e. if a particular correlation $\left( u, v \right)$is unexpected), it must hold that (see (20)):

$\frac{{cov}_{S\times\mu}\left( M_{u},M_{v} \right) {cov}_{S}\left( X_{u},X_{v} \right)}{\left( {cov}_{\mu}\left( \xi_{u},\xi_{v} \right) \right)^{2}}=\left( \frac{{cov}_{S}\left( X_{u},X_{v} \right)}{{cov}_{\mu}\left( \xi_{u},\xi_{v} \right)} \right)^{2}+\frac{{cov}_{S}\left( X_{u},X_{v} \right)}{{cov}_{\mu}\left( \xi_{u},\xi_{v} \right)}<0$. ( 23 )

This condition is of the form$R^{2}+R<0$, where$R=\frac{{cov}_{S}\left( X_{u},X_{v} \right)}{{cov}_{\mu}\left( \xi_{u},\xi_{v} \right)}$, which trivially has the solution:

$\frac{1}{R}=\frac{{cov}_{\mu}\left( \xi_{u},\xi_{v} \right)}{{cov}_{S}\left( X_{u},X_{v} \right)}< -1$. ( 24 )

The resulting inequality is satisfied under two conditions, which are thus requisite for a correlation to be unexpected, namely:

${|cov}_{\mu}\left( \xi_{u},\xi_{v} \right)\left| >{|cov}_{S}\left( X_{u},X_{v} \right) \right|$ ( 25 )

${cov}_{\mu}\left( \xi_{u},\xi_{v} \right){cov}_{S}\left( X_{u},X_{v} \right)<0$ ( 26 )

The first condition (25) is interpreted as a drowning out of the causal link between two nodes by error; that is, the magnitude of error in the correlation between two nodes’ expressions is greater than the magnitude of real correlation between them. The second condition (26) is interpreted as a counteracting of error to causal connections: the contribution to the empirical correlation between two nodes due to error must counteract the contribution due to causal mechanisms.

Condition (26) implies that, given the condition (22) for error distribution, PUC will statistically detect 50% of total false correlations for which the causal contribution is negligibly small, as the signs of the error and causal contribution are equally likely to be the same as they are to be opposite.

**II.4. Unexpected correlations under non-monotonicity.**

Here we prove the proposition in the conclusion about non-monotonic links. The statement says that a non-monotonic link between two nodes with an unexpected correlation cannot cause a transition between two distinct states of a network. We provide an extreme example of non-monotonicity, in which the dependence between two nodes changes in sign in the two states of a network (e.g. stimulation in one state of a biological system and inhibition in the other).

Assume we are given $n+2$ gene expressions in two biological state $P$ and $Q$: $X_{P}, Y_{P}, X_{1,P}, \ldots, X_{n,P}$ and $X_{Q}, Y_{Q}, X_{1,Q}, \ldots, X_{n,Q}$. We assume linear (or almost linear) dependence of $Y$ on $X$ within any one given biological state, stated as follows: $Y_{P}=\alpha_{P}X_{P}+\xi_{P}$ and $Y_{Q}=\alpha_{Q}X_{Q}+\xi_{Q}$, where $\xi_{P}$ is a function of $X_{1,P}, \ldots, X_{n,P}$, and $\xi_{Q}$ is a function of $X_{1,Q}, \ldots, X_{n,Q}$, and $\alpha_{P}\alpha_{Q}\neq0$. We suppose that $X_{P}$ ($X_{Q})$ and $\xi_{P}$ ($\xi_{Q}$) are independent. Recall that all gene expression values are positive and remember that $\Delta X:=\mathbb{E}_{P}\left[ X \right]-\mathbb{E}_{Q}\left[ X \right]\mathbb{=E[}X_{P}\mathbb{]-E[}X_{Q}]$.

*Lemma 4. Suppose* $\alpha_{P}\alpha_{Q}<0$ *(implying that the relation between X and Y is non-monotonic), then:*

1. $X$ *and* $Y$ *have unexpected correlations.*
2. *The sign of* $\Delta Y$ *may not depend on the sign of* $\Delta X$*, but instead mostly depends on the sign of* $\Delta\xi$*.*

*Proof*. Observe that, due to independence of $X_{P}$ ($X_{Q})$ and $\xi_{P}$ ($\xi_{Q}$):

${cov}_{P}\left( X,Y \right)=cov\left( X_{P},Y_{P} \right)=\alpha_{P}Var[X_{P}]$, ( 27 )

${cov}_{Q}\left( X,Y \right)=cov\left( X_{Q},Y_{Q} \right)=\alpha_{Q}Var[X_{Q}]$. ( 28 )

Therefore, ${cov}_{P}\left( X,Y \right){cov}_{Q}\left( X,Y \right)<0$ (so that the expected correlation inequalities do not hold simultaneously) if and only if $\alpha_{P}\alpha_{Q}<0$. This proves the item (a) of the lemma.

Let us prove (b). Without loss of generality, $cov_{P}\left( X,Y \right)<0$, implying $\alpha_{P}<0$ and $\alpha_{Q}>0$. Hence:

$\Delta Y=\mathbb{E}\left( Y_{P}-Y_{Q} \right)=\mathbb{E}\left( {\alpha_{P}X}_{P}-{\alpha_{Q}X}_{Q} \right)+\mathbb{E}(\xi_{P}-\xi_{Q})$ ( 29 )

Note that $\mathbb{E}\left( {\alpha_{P}X}_{P}-{\alpha_{Q}X}_{Q} \right)<0$ regardless of the values of $X_{P}$ and $X_{Q}$ (both of which are strictly positive). Thus in the case $\Delta\xi>0$ the change $\Delta Y$ will still be negative. The sign of $\Delta Y$ will be positive only if $\Delta\xi\gg0$. ☐

**III. Simulations using GeneNetWeaver.**

We tested PUC using GeneNetWeaver (GNW), a software package designed for rigorous testing of gene network inference methods. We used GNW to generate various networks ranging in size from 40 to 740 nodes, each broken into two disjoint subnetworks in a similar manner as with the previous simulations. Distinct equilibrium network states were made by performing a 50% knockdown on the node in each subnetwork with the most connections. Networks were simulated 100 times both stochastically and analytically. In the case of analytic simulations, in order to get distinct equilibria in different simulations all genes were given normally distributed microperturbations, i.e. proportional up/down regulations with mean 0 and a standard deviation of 1.25%. After each simulation, we selected those genes which were differentially expressed with FDR < 0.01%, and calculated correlations between them in each class separately. We computed PUC and true error for the resulting regulatory networks consisting of at least 20 nodes at various FDR cutoffs. The results are summarized in figures S3 a,b

1. b)


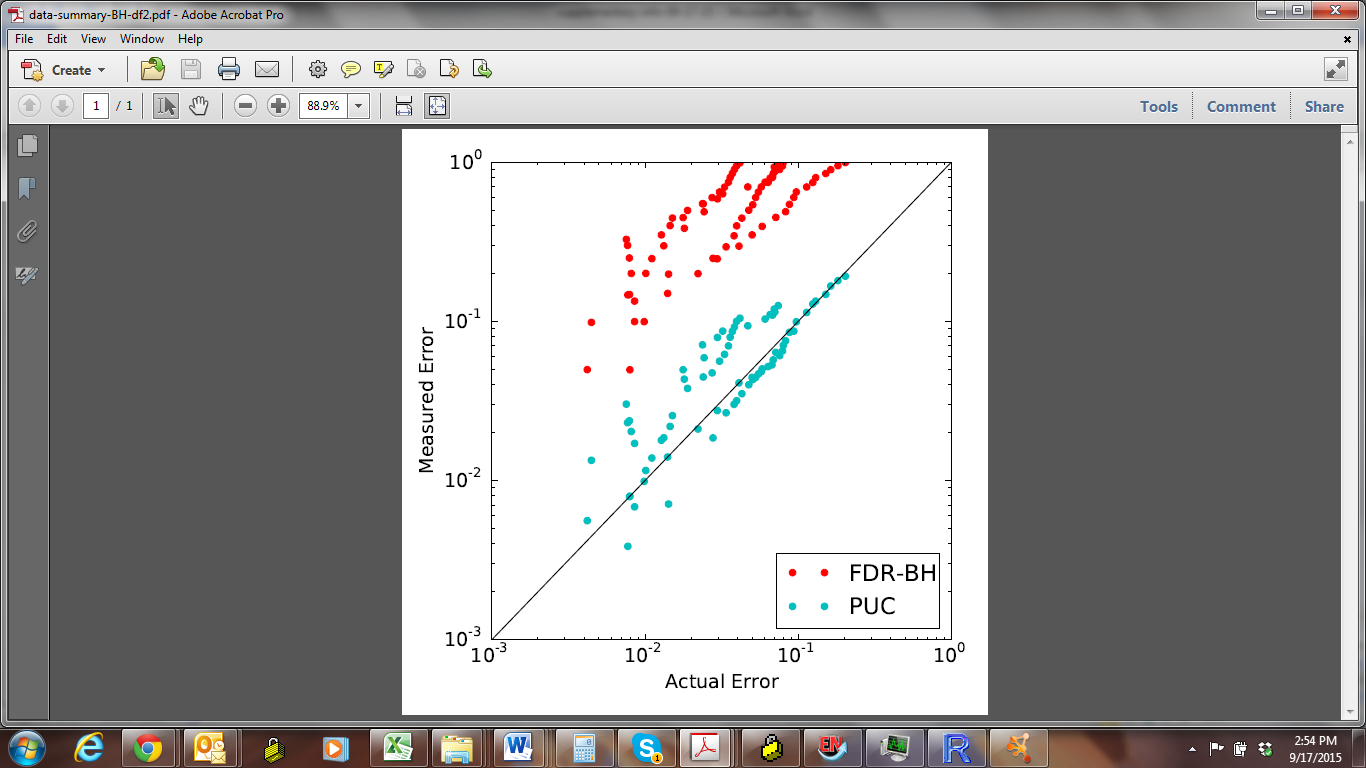

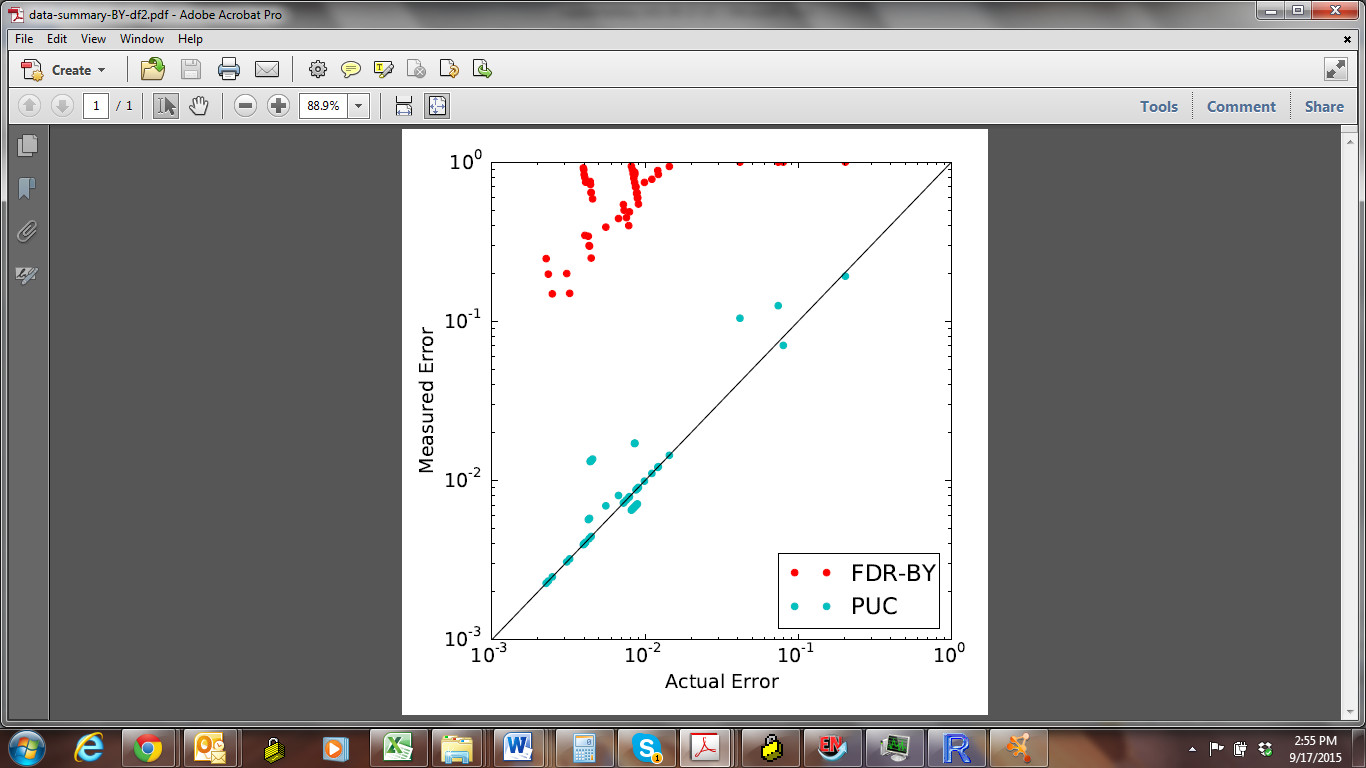


Figure S3. Comparison between PUC and FDR in networks simulated by GNW.

x axes represent actual error; y axes actual error (black line), PUC- blue dots, FDR –red dots (Benjamini-Hochberg- left panel; Benjamini-Yekutieli- right panel).

Figure S4. PUC, FDR-BH (a,c), FDR-BY (b,d) and error in networks of different sizes (number of nodes) simulated by GNW. Panels a) and b) show values for each metric (PUC, FDR or error). Panels c) and d) show the distance from error for FDR and PUC. Overall, PUC is closer to error than FDR.

1. b)

c) d)

**List of datasets from BRB Array Tools Archive used for analysis:**

GEO IDs:

GDS1021, GDS232, GDS408, GDS470, GDS484, GDS507, GDS531, GDS535, GDS536, GDS619, GDS690, GDS715, GDS760, GDS806, GDS838, GDS845-8, GDS884, GDS971, GDS978.

Note: for datasets that we could not find GEO ID we provide PUBMED IDs. All datasets were downloaded from BRB Array Tools Archive.

PUBMED IDs:

PMID:10359783, PMID:11707567, PMID:12925757, PMID:15548776, PMID:11707590.
